# Survival of *Rickettsia conorii* in artificially contaminated whole and leukoreduced canine blood units during the storage period

**DOI:** 10.1186/s13071-020-3991-9

**Published:** 2020-04-21

**Authors:** Laura Lucchese, Silvia Ravagnan, Graziana Da Rold, Federica Toniolo, Wendy Wurzburger, Monica Mion, Antonio Carminato, Pierre-Edouard Fournier, Gioia Capelli, Alda Natale, Marta Vascellari

**Affiliations:** 1 Istituto Zooprofilattico Sperimentale delle Venezie, Legnaro, PD Italy; 2 Centre National de Référence des Rickettsia, Coxiella, Bartonella, Marseille, France

**Keywords:** Leukoreduction, Blood transfusion, *Rickettsia conorii*, Dog

## Abstract

**Background:**

The ability of tick-borne agents to survive in stored blood bags is a key factor for their transmissibility by blood transfusion. The aim of this study was to evaluate the survival and potential infectivity of *Rickettsia conorii* (RC) in artificially contaminated canine whole blood (WB) and in leukoreduced whole blood (LR-WB) during the storage period.

**Methods:**

RC was cultured on L929 cells. We used a one-week 25-cm^2^ flask with 70–80% of L929 infected cells to prepare the bacterial inoculum by pelleting cells and suspending the pellet in the donors’ serum. We infected five 100 ml WB units with RC within 2 h from the collection and maintained it at room temperature for 4 h prior to refrigeration. We filtered 50 ml of each WB bag to obtain leukoreduced WB (LR-WB) at day 1 post-infection (dpi). We checked WB and LR-WB bags at 1, 4, 7, 14, 21, 28, 35 dpi for RC presence and viability through real-time PCR (rPCR) for DNA and mRNA, respectively, and by isolation. Identification of isolates was confirmed by indirect immunofluorescence and rPCRs.

**Results:**

RC survived for the entire storage period in both whole and leukoreduced blood. All bags contained viable bacteria until 7 dpi; RC viability generally decreased over time, particularly in LR-WB bags where the isolation time was longer than in WB. Viable bacteria were still isolated at 35 dpi in 3 WB and 3 LR-WB.

**Conclusions:**

Leukoreduction reduced but did not eliminate RC in infected units. The survival and infectivity of RC in canine blood during the storage period may represent a threat for recipients.
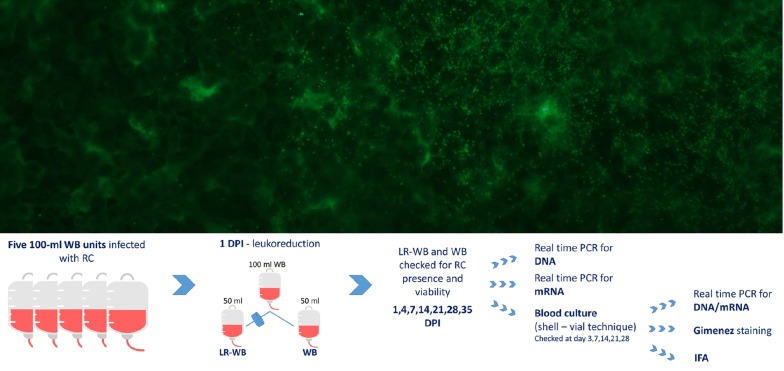

## Background

Transfusion medicine has acquired increasing importance in veterinary practice during the last decades and the establishment of blood banks, particularly for dogs, has led to a wider availability and use of blood components in life-saving therapy as well as the need of adequate screening tests for donors to minimize the risk of pathogen transmission by transfusion.

Among infectious agents, those transmitted by ticks are critical threats in transfusion medicine because of their transmissibility by blood. *Ehrlichia* spp., *Babesia* spp. and *Anaplasma* spp. are typically included in recommended testing for canine donors, but other pathogens may be assessed on the basis of geographical distribution and clinical significance [1]. Several *Rickettsia* spp. belonging to the spotted fever group are present in southern Europe and are transmitted by tick bites [2]. In particular, *Rickettsia conorii* (RC), the causative agent of Mediterranean spotted fever in humans, is transmitted by the brown dog tick, *Rhipicephalus sanguineus*, its main vector and possible reservoir in Europe [3]. This species has been shown to be experimentally transmitted to dogs by ticks [4]. Due to the presence of clinical symptoms of illness observed in experimentally infected dogs [4, 5], as well as the occurrence of sporadic cases of natural infection [6, 7], it is recommended to include this pathogen in the screening panel for canine blood donors.

The intracellular location of RC, together with the non-specific and sometimes subclinical course of the disease, represent a key factor to assume the RC potential transfusion risk, even though no data regarding the survival and infectivity of RC during the blood storage period is available.

In human medicine, several tick-borne pathogens with intracellular location have raised concerns because of the risks they pose to blood safety [8]. *Anaplasma phagocytophilum* [9] and *Babesia microti* [10] have shown to be capable of surviving 18–21 days in stored blood conditions. Furthermore, it is reported that they caused transfusion cases in humans even later than the survival demonstrated in laboratory experiments, suggesting a possible longer transmissibility by infected units [11]. Despite the relevance of this issue, information on survival of many pathogens is still undefined and often based on the age of the blood that transmitted the infection [8]. *Rickettsia rickettsii* has been suspected to survive at least 9 days in stored blood, because the only transfusion case described acquired the infection from a 9-day refrigerated whole blood unit [12].

Leukoreduction is a common practice in human transfusion medicine, thanks to the use of in-line filters integrated in the bag system, which allow the reduction of the white blood cell count by 99.9 % [13]. The primary effect of leukoreduction is to limit storage lesions, due to leukocyte degradation during storage; a secondary effect could be to remove white blood cells potentially infected by intracellular infectious agents, such as rickettsiae, which can be transmitted to the recipient by the blood transfusion. Leukoreduction seemed to reduce the risk of transfusion-transmitted infections in laboratory models using *Orientia tsutsugamushi* [14] and *Anaplasma phagocytophilum* [15], even though pathogens were not completely eliminated from blood units.

Even if leukoreduction is suggested to reduce the risk of transmission of different blood pathogens, the efficacy in eliminating RC in infected units and the effect on RC viability under storage conditions are still undetermined.

The aim of this study was to evaluate the survival and the potential infectivity of RC in canine whole blood (WB) and in leukoreduced whole blood (LR-WB) during a 35-day storage period.

## Methods

### Whole blood unit collection

Canine donors were selected among healthy, privately-owned dogs included in the blood donor programme of the IZSVe canine blood bank. The donors fulfilled the following inclusion criteria: age 2–8 years; body weight ≥ 25 kg; clinically healthy; regularly vaccinated and protected against endo- and ectoparasites, according to the Italian Ministry of Health guidelines [16]. At each donation, donors underwent general hematological and biochemical screening, as well as serological and biomolecular tests against vector-borne pathogens (*Leishmania* spp., *Rickettsia* spp., *Ehrlichia canis*, *Anaplasma phagocytophilum* and *Babesia canis*). For ethical reasons and considering the clinical importance of the blood unit supply, no more than five registered donors were included in the study. The dogs’ owners formally approved the use of part of the donation for research purposes before the sampling and they approved an information form. The donation was performed by a double bag collection system, equipped with an in-line integrated leukocyte reduction filter from the jugular vein after trichotomy and accurate disinfection of the site. The collection bag contained 14 ml of citrate phosphate-dextrose-adenine (CPDA-1) to collect 100 ml of WB (ratio1:7). The bag system was also equipped with a self-cleaning valve that allowed the collection of blood samples while maintaining the sterility during the study period.

WB units were inoculated within 2 h from collection, keeping the bags at room temperature to preserve leukocyte viability. Before infection, 1.5 ml of WB were collected from each unit to guarantee the negativity of the WB unit for RC (rPCRs and blood culture on L929 as described below).

### RC culture and blood unit infection

RC was cultured at 32 °C in 5% CO_2_ and 95% humidity on L929 cells, a murine fibroblastic cell line suitable for RC isolation and maintenance [17, 18], with MEM enriched with 4% bovine fetal serum and 1% of l-glutamine. L929 cells cultured for one-week in a 25-cm^2^ flask until confluent monolayer were infected with RC and the strain was propagated every week in new 25-cm^2^ flasks using serial inoculum dilutions until infection stabilization to obtain a 25-cm^2^ flask with a monolayer containing 70–80% of infected cells every week. The cell infection rate was estimated by Gimenez staining on a sample of cells collected from the monolayer and stained on a glass slide. The RC dilution for the inoculum was set according to the limit of detection (LOD) of the real-time PCR assay targeting DNA of RC (assay described below), in order to obtain a WB bag infection rate at the LOD level (10 DNA copies /µl). This was achieved using the entire RC production of a 1-week 25-cm^2^ flask to infect 100 ml of WB. The inoculum was prepared after mechanical detachment and partial needle (27 G) separation by pelleting cells (centrifugation at 10000×*g* for 10 min), removal of the supernatant followed by two washes of the pellet with sterile PBS and by re-suspending the pellet in 1.2 ml of donor’s serum, obtaining 1 ml for unit infection and 200 µl for molecular analyses. One drop (*c.*10 µl) was examined by Gimenez staining.

WB units were infected with the RC inoculum through the self-cleaning valve using a sterile syringe; the bags were gently shaken to favor the distribution of the infected cells and free RC and kept at room temperature for 2–4 h to allow infection stabilization. Then, all units were stored at 4 °C ± 2, simulating ordinary storage conditions. At day 1 post-infection (dpi), half of the WB was filtered for leukoreduction and collected in the secondary bag (LR-WB). Both the WB and LR-WB units were stored at 4 °C ± 2 for 35 days (unit shelf life).

### Infection assessment

**Fig. 1:**
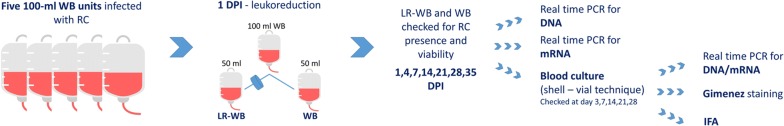
Graphical representation of the procedure used in this study. *Abbreviations*: WB, whole blood; LR-WB, leukoreduced whole blood; dpi, day post-infection; RC, *Rickettsia conorii*

At 1, 4, 7, 14, 21, 28 and 35 dpi, 1.2 ml of blood were sampled from all WB and LR-WB units and tested through rPCRs for DNA and RNA (200 µl) and by culture (1 ml) (Fig. 1) to assess RC presence and viability.

#### Molecular analyses

Total DNA and RNA were extracted from 200 µl of blood or culture supernatant, using an automated extractor and the MagMax DNA/RNA pathogen kit, according to the manufacturer’s instructions (Thermo Fisher Scientific, Waltham, MA, USA). Real-time PCR (rPCR) was used to test RC DNA presence, while RT (reverse transcriptase) rPCR for mRNA was used as a viability marker of the bacterium on rPCR-positive samples.

DNA was amplified using a SYBR green real-time PCR assay performed with the primers RompB OF (5′-GTA ACC GGA ART AAT CGT TTC GT-3′) and RompB OR (5′-GCT TTA TAA CCA GCT AAA CCR CC-3′), targeting 511 bp of the *ompB* gene [19]. The reaction mix contained 10 µl of QuantiFast SYBR green PCR master mix (Qiagen, Hilden, Germany) (1×), 0.2 µl of forward and reverse primers (0.1 µM), 3 µl of DNA and RNase-free H_2_O up to a final volume of 20 µl. The thermal profile consisted of 95 °C for 5 min, followed by 40 cycles at 95 °C for 15 s, 58 °C for 30 s and 60 °C for 30 s. Following amplification, melt curve analysis was performed by slowly raising the temperature of the thermal chamber from 60 °C to 95 °C to distinguish between specific and non-specific amplification products.

A RT real-time PCR assay was applied to mRNA, using the primers Rick-mRNA groEL F (5’-TCC ATA CCG CCC ATA CCT CCC A-3’), and Rick-mRNA groEL R (5’-AGA TGC TGC TTC CGT TGC TTC G-3’) and the Rick-mRNA groEL P probe (5’-CCG CCA CGC ATT GGC ATT GGC TCT GCC-3’), targeting 119 bp of the *groEL* gene [20]. The reaction mix contained 10 µl of RT-rPCR master mix (Qiagen, Hilden, Germany) (1×), 0.6 µl of forward and reverse primers (0.3µM), 0.2 µl of enzyme mix, 3 µl of mRNA and RNase-free H_2_O up to a final volume of 20 µl. The thermal profile consisted of 20 min at 50 °C, 15 min at 95 °C, followed by 45 cycles at 94 °C for 30 s and 60 °C for 1 min.

For each DNA and mRNA amplification reactions, a negative (H_2_O) and positive (*Rickettsia* spp.) controls were included.

All *ompB* rPCR products were sequenced, using a 16-capillary ABI PRISM 3130xl Genetic Analyzer (Applied Biosystems, Foster City, CA, USA), to confirm the positivity for RC. Sequence data were assembled and edited with SeqScape software v2.5 (Applied Biosystems). Sequences obtained were aligned and compared with representative sequences available on GenBank.

#### Blood culture

Isolation of RC from infected WBs and LR-WBs was performed using the shell-vial technique [21] and L929 cells. For each bag, five shell vial tubes containing a 1 cm round slide with a confluent L929 cell monolayer and 1 ml of culture medium were inoculated with 200 µl of blood. After 1 h of centrifugation at a low speed (700× *g*) to favor cell infection, the culture medium containing blood was discarded and replaced with 1 ml of fresh medium. Tubes were incubated at 32 °C, 5% CO_2_ and 95% humidity. Bacterial growth was assessed after 3, 7, 14, 21 and 28 days by processing one of the tubes, while the remaining tubes were kept for the following culture monitoring, changing culture medium once a week until day 28 or RC isolation. At every follow-up examination, after collecting a small portion of the infected cell monolayer, the culture supernatant was removed, one drop was put on a slide, dried and stained with the Gimenez method; 200 µl of supernatant was tested by rPCR as described for WB and LR-WB samples. The slide with the L929 cell monolayer was fixed with methanol and tested by IFA for the presence of RC as follows: the slide was transferred in a 1 cm round well with 300 µl of RC positive dog serum diluted 1:64 and incubated for 30 min at 37 °C in a humid chamber. After two washes with sterile PBS, 300 µl of anti-dog fluorescein-labeled antibody (Sigma-Aldrich, St Louis, Missouri, USA) was added and the slide incubated for 30 min at 37 °C in a humid chamber, washed twice with PBS and read using a fluorescence microscope. Each negative control tube containing an uninfected L929 monolayer was maintained and tested in the same conditions as the RC culture tubes.

#### Viability criteria

RC viability in blood samples was considered confirmed when rPCR for mRNA and/or culture gave a positive result. The blood culture test was considered positive when RC was identified by RT-rPCR from infected cells and/or IFA. The identification of intracellular RC by Gimenez staining without positivity to other tests was not considered indicative of RC isolation. The positivity of rPCR-DNA only from both blood and culture supernatant was considered indicative of RC presence, but not viability.

### Statistical analysis

The analysis of variance (ANOVA) was used to compare the average time (in days) of the first positive culture from samples of WB and LR-WB, recorded through the study at each dpi. The agreement among the test used in culture to assess viability of RC in the stored blood units (i.e. rPCR, Gimenez stain and IF) was evaluated using the kappa (*k*) coefficient. The value of *k* varies from 0 (perfect discordance) to 1 (perfect concordance) and its interpretation was given as follows: weak (0.1–0.2); moderate (0.20–0.4); discrete (0.4–0.6); substantial (0.6–0.8); and very good concordance (0.8–1.0) [22]. The software used for statistical analysis was SPSS for Windows, version 13.0.

## Results

All rPCR tests and WB culture from the five blood units before infection gave negative results. The RC inoculum was rPCR-positive in all cases before infection and Gimenez staining of RC culture showed the expected rate of infection. Detailed results of the tests performed in the blood units and on the supernatant samples from the cultures at every dpi are reported in Additional file 1: Table S1.

**Fig. 2:**
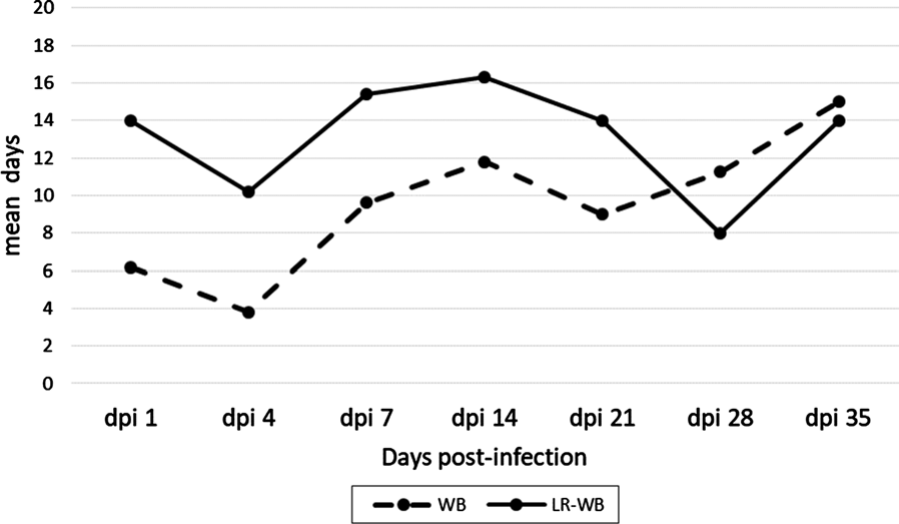
Mean number of days required to obtain positivity for RC on culture from blood collected at days post-infection (dpi) 1, 4, 7, 14, 21, 28 and 35 in whole blood units (WB) and leukoreduced blood units (LR-WB)

At 1 dpi, all five WB units were RT-rPCR-positive for viable RC, and 2/5 LR-WB units were rPCR-positive only, with no detection of mRNA (Table 1). However, cultures from all five LR-WBs at 1 dpi became positive two weeks later (day 14), showing that a certain amount of viable RC successfully passed through the filter (Table 2). Viable RCs were detectable in all five WB units until 21 dpi and in all five LR-WB units until 7 dpi; three units of both WB and LR-WB had viable RC until 35 dpi. LR-WB required a higher mean number of days to obtain a positive culture compared to WB (Fig. 2) (ANOVA: *F*_(32, 28)_ = 5.911, *P* = 0.018), until 21 dpi, suggesting a lower burden in LR-WB units.

**Table 1:** Results of rPCR-DNA and of RT-rPCR performed on the samples from the blood units at different days post-infection (dpi), in whole blood units (WB) and leukoreduced blood units (LR-WB)

PCR blood	WB units					LR-WB units				
(dpi)	A	B	C	D	E	A	B	C	D	E
1	POS/v	POS/v	POS/v	POS/v	POS/v	POS/nv	neg	neg	neg	POS/nv
4	POS/v	neg	POS/nv	POS^a^	neg	POS/nv	neg	POS/nv	neg	POS/nv
7	POS/v	POS^a^	neg	POS/v	POS/v	POS/nv	neg	neg	neg	neg
14	POS/v	neg	POS/nv	POS/v	neg	POS/nv	neg	neg	neg	neg
21	POS/v	neg	neg	neg	POS/v	neg	neg	neg	neg	POS/nv
28	POS/v	POS/nv	POS/nv	POS/v	neg	POS/nv	neg	neg	neg	neg
35	POS/v	neg	neg	neg	POS/v	POS/nv	neg	neg	neg	POS/v

^a^Not tested by RT-rPCR

*Abbreviations*: v, viable bacteria; nv, non-viable bacteria; POS/nv, positive rPCR only; POS/v, positive RT-rPCR

**Table 2:** Day (3, 7, 14, 21 and 28) of the culture positivity from samples taken at each day post-infection in whole blood (WB) and leukoreduced blood (LR-WB) units

Day post-infection	Day of isolation									
	Unit A		Unit B		Unit C		Unit D		Unit E	
	WB	LR WB	WB	LR WB	WB	LR WB	WB	LR WB	WB	LR WB
1	7	14	7	14	3	14	7	14	7	14
4	3	3	3	14	3	3	7	28	3	3
7	3	14	14	14	3	7	21	28	7	14
14	14	neg	14	14	3	14	14	neg	14	21
21	14	21	14	14	3	7	7	neg	7	14
28	21	3	7	7	3	neg	neg	neg	14	14
35	28	14	14	neg	ne	14	ne	neg	3	14

*Abbreviations*: ne, not examined; neg, negative

Considering the combined results of the tests performed on blood and cultures, all WB and LR-WB blood units were classified positive, either by RT-rPCR from blood and/or evidence of growth in culture.

Tests on blood culture showed a substantial agreement in identifying RC, with a few exceptions: in 3 cases, IFA tested positive before rPCRs; and in 7 cases, mRNA was detected before IFA was positive (Additional file 1: Table S1). In more detail, the *k* coefficient (measure of concordance) was 0.698 between rPCR and IFA, 0.626 between rPCR and Gimenez stain and 0.761 between Gimenez stain and IFA.

For two blood cultures, the Gimenez staining allowed detecting the presence of intracellular RC before IFA or rPCRs. In only one case, the detection of RC by Gimenez staining in a LR-WB at 4 dpi was not confirmed by any other test. Neither evidence of aspecific L929 cytopathic effect nor bacterial/yeast culture contamination was registered by microscopic cell observation during the experiment and negative control tubes always tested negative.

## Discussion

This study highlighted that leukoreduction reduced but did not eliminate RC in infected units. RC could still remain viable at 4 °C during the storage period when RC passed the blood filter, even if no evidence of RC replication was obtained.

No previous data are available regarding the survival and possible replication of RC in stored blood units. Since RC is an obligate intracellular bacterium, it was not expected to survive during the entire storage period. Unexpectedly, this study demonstrated that RC was able to remain viable in WB stored until 35 days. Moreover, we demonstrated that, despite leukoreduction can eliminate almost all circulating cells that could support RC survival, LR-WB maintained a potential RC infectivity during the entire storage period.

*Coxiella burnetii*, another obligate intracellular gram-negative bacterium, was demonstrated to survive free in stored blood units and blood products until 45 dpi [23], but the environmental stability and the capacity of *C. burnetii* to form a spore-like variant [24] are not described for RC. Long-term survival was also reported for *A. phagocytophilum*, with a viability in blood observed until day 18 at 4 °C [9]. In addition, viable bacteria were detected after leukoreduction in blood units experimentally spiked with infected cells [15]. However, the duration of *A. phagocytophilum* survival in LR-WB was not determined.

The storage temperature may have influenced RC survival and partially explains the findings of this study. In fact, the optimal culture and maintenance temperature for RC is 32 °C [25]; we assume that the blood storage temperature of 4 °C may have limited RC replication, maintaining RC viable but in a quiescent state, still able to infect cells in favourable culture conditions. Thus, the low temperature and the blood environment may favour the long-term survival of RC, even in absence of cells suitable to support bacterial replication.

This study had some limitations: (i) RC bacteria in the inoculum were not quantified precisely, but estimated based on the LOD of the PCR used, and this may have caused some variability among results of the five units; and (ii) the mechanical detachment of L929 infected cells for inoculum preparation may have induced the lysis of a portion of cells and the release of free RC, reducing the ability of the filter to stop RC.

Apart the possible bias related to the experimental model, RC growth in LR-WB took longer than in WB, supporting the effect of leukoreduction to decrease the bacterial load. For the purpose of the study, the initial bacterial load was set on the LOD of the analytical method used (screening rPCR) and blood units were infected with a bacterial load at a level barely detectable by rPCR. This situation may simulate the field case of clinically healthy donor dogs, coming to donation with an ongoing subclinical infection and low bacterial load that could escape routine checks. RC circulating in the blood in an intracellular form (endothelial cells) or, to a minimum part free, can remain viable during the storage period and can potentially be transmitted to a recipient by blood transfusion. Little is known about the course of RC or *Rickettsia* spp. infection in dogs. Usually, RC infection is characterized by transient mild symptoms or subclinical presentation [6, 7], which is confirmed by experimental infections [4, 5]. Despite the high *Rickettsia* spp. seroprevalence reported in some regions [26, 27], clinical cases of RC in dogs are sporadic [6, 7], supporting the hypothesis of the low pathogenicity of RC for dogs. However, the transmission of RC to a critically-ill recipient with a compromised immune system would make this infection more severe.

For all these reasons, RC should be included in the screening panel of canine blood donors. However based on the results of this study, it is preferable to maintain a high standard of selection of healthy blood donors, in particular focusing on the prevention of tick-bites, rather than relying on the sensitivity of the laboratory methods used for screening.

## Conclusions

RC infection can be a severe clinical threat in immunocompromised transfused dog patients. Leukoreduction reduced the bacterial load linked to the presence of RC in blood units, but the presence of free RC remains a possible risk for the recipient subjects. Leukoreduced blood units may remain potentially infectious for the entire blood bag shelf life, even due to small quantities of RC not easily detectable. An integrated approach that combines prevention of tick-bites with recurrent serological and molecular screening is recommendable for donor monitoring to reduce the risk of transfusion-transmitted tick-borne infections.

## Supplementary information

**Additional file 1: Table S1.** Detailed results of the tests performed on blood units at different days post-infection (dpi) and on cultures from days 3 to 28. *Rickettsia conorii* was considered viable (v) or non-viable (nv), when the unit or the culture were positive/negative by RT-rPCR and/or immunofluorescence (IF).

## Data Availability

Data supporting the conclusions of this article are included within the article and its additional file.

## Abbreviations

RC: *Rickettsia conorii*
WB: whole blood
LR-WB: leukoreduced whole blood
dpi: day post-infection
CPDA-1: citrate phosphate-dextrose-adenine
LOD: limit of detection
rPCR: real-time polymerase chain reaction
RT-rPCR: reverse transcriptase real-time polymerase chain reaction
