# Supplementary material for: Survival of Rickettsia conorii in artificially contaminated whole and leukoreduced canine blood units during the storage period
Source: Parasit Vectors. 2020 Apr 21;13:118. doi: 10.1186/s13071-020-3991-9 (PMC7171768; doi:10.1186/s13071-020-3991-9)
Supplement: Supplementary file 1 — Additional file 1: Table S1. Detailed results of the tests performed on blood units at different days post-infection (dpi) and on cultures from days 3 to 28. Rickettsia conorii was considered viable (v) or non-viable (nv), when the unit or the culture were positive/negative by RT-rPCR and/or immunofluorescence (IF). [file 13071_2020_3991_MOESM1_ESM.docx]

**Additional file 1: Table S1.** Detailed results of the tests performed in blood units at different days post infection (DPI) and from cultures from days 3 to 28. *Rickettsia conorii* was considered viable (v) or not viable (nv), when the unit or the culture were positive/negative by RT-rPCR and/or immunofluorescence (IF).

| **Unit** | **DPI** |  | **PCRs (blood)** | **Results of PCRs, Gimenez stain (G) and Immunofluorescence (IF) on culture** | | | | |
| --- | --- | --- | --- | --- | --- | --- | --- | --- |
|  |  |  |  | **day 3** | **day 7** | **day 14** | **day 21** | **day 28** |
| **A** | D1 | WB | POS/v | neg | PCR/IF (v) |  |  |  |
| **A** | D1 | LR-WB | POS/nv | neg | neg | PCR/G/IF (v) |  |  |
| **A** | D4 | WB | POS/v | IF (v) | PCR/G/IF (v) |  |  |  |
| **A** | D4 | LR-WB | POS/nv | IF (v) | PCR/IF (v) | PCR (v) |  |  |
| **A** | D7 | WB | POS/v | PCR/IF (v) | PCR/G (v) | PCR (v) |  |  |
| **A** | D7 | LR-WB | POS/nv | neg | neg | PCR/G/IF (v) |  |  |
| **A** | D14 | WB | POS/v | neg | neg | PCR/G/IF (v) |  |  |
| **A** | D14 | LR-WB | POS/nv | neg | neg | neg | neg | neg |
| **A** | D21 | WB | POS/v | neg | neg | PCR/G/IF (v) |  |  |
| **A** | D21 | LR-WB | neg | neg | neg | neg | PCR/G/IF (v) |  |
| **A** | D28 | WB | POS/v | neg | neg | neg | PCR/G/IF (v) |  |
| **A** | D28 | LR-WB | POS/nv | PCR/IF (v) | neg | neg | neg | neg |
| **A** | D35 | WB | POS/v | neg | neg | neg | neg | PCR/IF (v) |
| **A** | D35 | LR-WB | POS/nv | neg | neg | PCR/G/IF (v) |  |  |
| **B** | D1 | WB | POS | neg | PCR/IF (v) | neg | PCR (v) |  |
| **B** | D1 | LR-WB | neg | neg | neg | PCR/IF (v) |  |  |
| **B** | D4 | WB | neg | PCR/G/IF (v) |  |  |  |  |
| **B** | D4 | LR-WB | neg | neg | neg | PCR/G/IF (v) |  |  |
| **B** | D7 | WB | POS | neg | neg | PCR/G/IF (v) |  |  |
| **B** | D7 | LR-WB | neg | neg | neg | PCR/G/IF (v) |  |  |
| **B** | D14 | WB | neg | neg | neg | PCR/G/IF (v) |  |  |
| **B** | D14 | LR-WB | neg | neg | neg | PCR/G/IF (v) |  |  |
| **B** | D21 | WB | neg | neg | neg | POS (v) | PCR/G/IF (v) |  |
| **B** | D21 | LR-WB | neg | neg | neg | POS (v) | PCR/G/IF (v) |  |
| **B** | D28 | WB | POS/nv | neg | G | neg | PCR/G/IF (v) |  |
| **B** | D28 | LR-WB | neg | neg | G | PCR (v) | neg |  |
| **B** | D35 | WB | neg | neg | neg | PCR/G/IF (v) |  |  |
| **B** | D35 | LR-WB | neg | neg | neg | neg | neg | neg |
| **C** | D1 | WB | POS/v | IF (v) | neg |  |  |  |
| **C** | D1 | LR-WB | neg | neg | neg | PCR/G/IF (v) |  |  |
| **C** | D4 | WB | POS/nv | PCR/G/IF (v) | PCR/G/IF (v) |  |  |  |
| **C** | D4 | LR-WB | POS/nv | G | neg | neg |  |  |
| **C** | D7 | WB | neg | G/IF (v) | PCR (v) |  |  |  |
| **C** | D7 | LR-WB | neg | neg | neg/G/IF |  |  |  |
| **C** | D14 | WB | POS/nv | G/IF (v) | PCR (v) |  |  |  |
| **C** | D14 | LR-WB | neg | neg | neg | PCR/G/IF (v) | PCR (v) |  |
| **C** | D21 | WB | neg | PCR/G/IF (v) | PCR/IF (v) |  |  |  |
| **C** | D21 | LR-WB | neg | neg | PCR (v) | POS/v/IF |  |  |
| **C** | D28 | WB | POS/nv | G/IF (v) | neg |  |  |  |
| **C** | D28 | LR-WB | neg | neg | neg | neg | neg |  |
| **C** | D35 | WB | neg | ne | ne | ne | ne | ne |
| **C** | D35 | LR-WB | neg | neg | neg | neg | POS/v/IF |  |
| **D** | D1 | WB | POS/v | neg | PCR/G/IF (v) | PCR (v) |  |  |
| **D** | D1 | LR-WB | neg | neg | neg | PCR/G/IF (v) |  |  |
| **D** | D4 | WB | POS | neg | PCR/G/IF (v) | PCR (v) |  |  |
| **D** | D4 | LR-WB | neg | neg | neg | neg | neg | PCR (v) |
| **D** | D7 | WB | POS/v | neg | neg | neg | PCR/G/IF (v) |  |
| **D** | D7 | LR-WB | neg | neg | neg | neg | neg | PCR/G/IF (v) |
| **D** | D14 | WB | POS/v | neg | neg | PCR/G/IF (v) |  |  |
| **D** | D14 | LR-WB | neg | neg | neg | neg | neg | neg |
| **D** | D21 | WB | neg | neg | PCR/IF (v) | PCR (v) |  |  |
| **D** | D21 | LR-WB | neg | neg | neg | neg | neg | neg |
| **D** | D28 | WB | POS/v | neg | neg | neg | neg | neg |
| **D** | D28 | LR-WB | neg | neg | neg | neg | neg | neg |
| **D** | D35 | WB | neg | ne | ne | ne | ne | ne |
| **D** | D35 | LR-WB | neg | neg | neg | neg | neg | neg |
| **E** | D1 | WB | POS/v | neg | PCR/G/IF (v) |  |  |  |
| **E** | D1 | LR-WB | POS | neg | neg | PCR (v) | PCR/G/IF (v) |  |
| **E** | D4 | WB | neg | PCR/G/IF (v) |  |  |  |  |
| **E** | D4 | LR-WB | POS/nv | PCR/G (nv) | PCR/G/IF (v) |  |  |  |
| **E** | D7 | WB | POS/v | neg | PCR/G/IF (v) |  |  |  |
| **E** | D7 | LR-WB | neg | neg | neg | PCR/G/IF (v) |  |  |
| **E** | D14 | WB | neg | neg | neg | PCR/G/IF (v) |  |  |
| **E** | D14 | LR-WB | neg | neg | neg | neg | PCR/G/IF (v) |  |
| **E** | D21 | WB | POS/v | neg | PCR/G/IF (v) | PCR (v) |  |  |
| **E** | D21 | LR-WB | POS/nv | neg | neg | PCR/G/IF (v) |  |  |
| **E** | D28 | WB | neg | neg | neg | PCR/G/IF (v) |  |  |
| **E** | D28 | LR-WB | neg | neg | neg | PCR/G (v) |  |  |
| **E** | D35 | WB | POS/v | PCR (v) | PCR/G/IF (v) |  |  |  |
| **E** | D35 | LR-WB | POS/v | neg | neg | PCR/G (v) |  |  |

ne= not examined
